# The vibrational response of simulated Ginkgo biloba fruit based on their frequency spectrum characteristics

**DOI:** 10.1371/journal.pone.0235494

**Published:** 2020-07-23

**Authors:** Yan Xuan, Linyun Xu, Guanhua Liu, Jie Zhou

**Affiliations:** College of Mechanical and Electronic Engineering, Nanjing Forestry University, Nanjing, Jiangsu, China; Northeastern University, UNITED STATES

## Abstract

The most effective method for harvesting forest fruit is the mechanical vibration harvesting method. During the forced vibration process, the fruit will be shed from the tree when the inertia of the fruit is greater than the fruit’s pedicel retention force. In order to study the movement response characteristics of the Ginkgo biloba fruit in depth, for a small Ginkgo biloba fruit tree, the frequency curve of the fruit tree had been obtained in this paper, based on the pulse hammer excitation method, and four resonant frequencies and four trough point frequencies, in the frequency range of 10 Hz~25 Hz, were determined as the test excitation frequency. Through a comparison test between the simulated fruit and the Ginkgo biloba fruit, both the simulated fruit and the real Ginkgo biloba fruit demonstrated good response consistency, and the results had shown that the simulated fruit could be used to replace the *Ginkgo biloba* fruit. The acceleration response of the resonant frequency and the trough point frequency for two test points of the two primary branches had also been analyzed. It was found that the resonant frequency caused an obvious harmonic response. For the same frequency, the fruit at some points produced a very strong vibrational response, while at other points the fruit was almost stationary. Therefore, it was difficult for a fruit tree to completely shed all its fruit through excitation at a single frequency. It was more difficult to induce a strong vibrational response of fruit on branches of higher stiffness. On the contrary, it was easier to induce a strong vibrational response on more flexible branches regardless of the resonant frequency or the trough point frequency excitation.

## 1. Introduction

Ginkgo biloba is also known as a deciduous tree that belongs to the genus Ginkgo of the family Ginkgoaceae; its fruit, which is rich in protein and fat, is very nutritious, and it also has medicinal properties. Ginkgo biloba trees cover an area in China of 1.2 billion hm^2^, and China accounts for 90% of the world’s production [1–4]. The best method for the mechanized harvesting of Ginkgo biloba is vibration harvesting, that is to say, an excitation device is clamped to the trunk to transfer the excitation energy from the excitation point of the trunk to the fruit, so that the vibrational response of the fruit is accelerated to produce an inertial force, and the fruit will then be shed when the inertial force of the fruit is greater than the binding force of the fruit’s stalk.

The mechanism of shedding the fruit and the dynamic response characteristics of the forced vibration of fruit had been studied around the world [5, 6]. The current research has theoretically established a fruit-stem response model and analyzed the parameters that affect the fruit’s dynamic response [7–10]. Torregrosa and Cai used high-speed cameras to study the movement of citrus fruit and apricots, and analyzed their response relationships to dynamic parameters such as displacement and acceleration [11, 12]. Wang et al studied the main factors that affect the removal rate of walnuts, grapes, apricots, citrus fruit and cherries, and they found that the frequency, amplitude and time had an effect on the removal rate [13–21]. Du et al analyzed the harvesting mechanism of cherries and pecans, and they carried out experimental research on the transmission of vibrational energy in fruit trees [22–24]. He et al [25] studied the dynamic transfer characteristics between Lycium chinense branches, and they obtained the acceleration response relationship between third branches and fourth branches. Fu et al [26] studied the force transmission effect of each offshoot of jujube, and they found a good force transmission effect when the amplitude was 7mm and the frequency was 17 Hz. Lin et al [27, 28] studied the frequency spectrum characteristics of Ginkgo biloba trees and they found that the optimal excitation frequency was 23.75 Hz to cause the fruit to be shed. Fruit generally has both long stalks and short stalks; both Ginkgo biloba and cherries are long-stalk fruit. The main movement state of long-stalk fruits was plane pendulum movement, rotation around the fruit and the fruit’s stalk connection point, reciprocating movement in the vertical direction, and circular motion around the axis of the fruit’s stalk in space.

In summary, the current research mainly focused on the fruit’s response at a set frequency and the correspondence between the excitation parameters and the rate of fruit shedding, however, research on the resonance response based on the frequency spectrum characteristics of fruit trees and the comparative relationship between the fruit’s movement patterns and the fruit’s dynamic response is still scarce. In this paper, especially for Ginkgo biloba fruit with long stalk characteristics, the frequency spectrum characteristics of the Ginkgo biloba tree have been analyzed, and the dynamic response and movement law of the fruit under resonant frequency excitation have been studied. The results of this work have provided a certain theoretical basis for an in-depth understanding of the fruit’s movement response and improving the fruit removal rate.

## 2. Materials and methods

### 2.1 Sample tree

**Fig 1:**
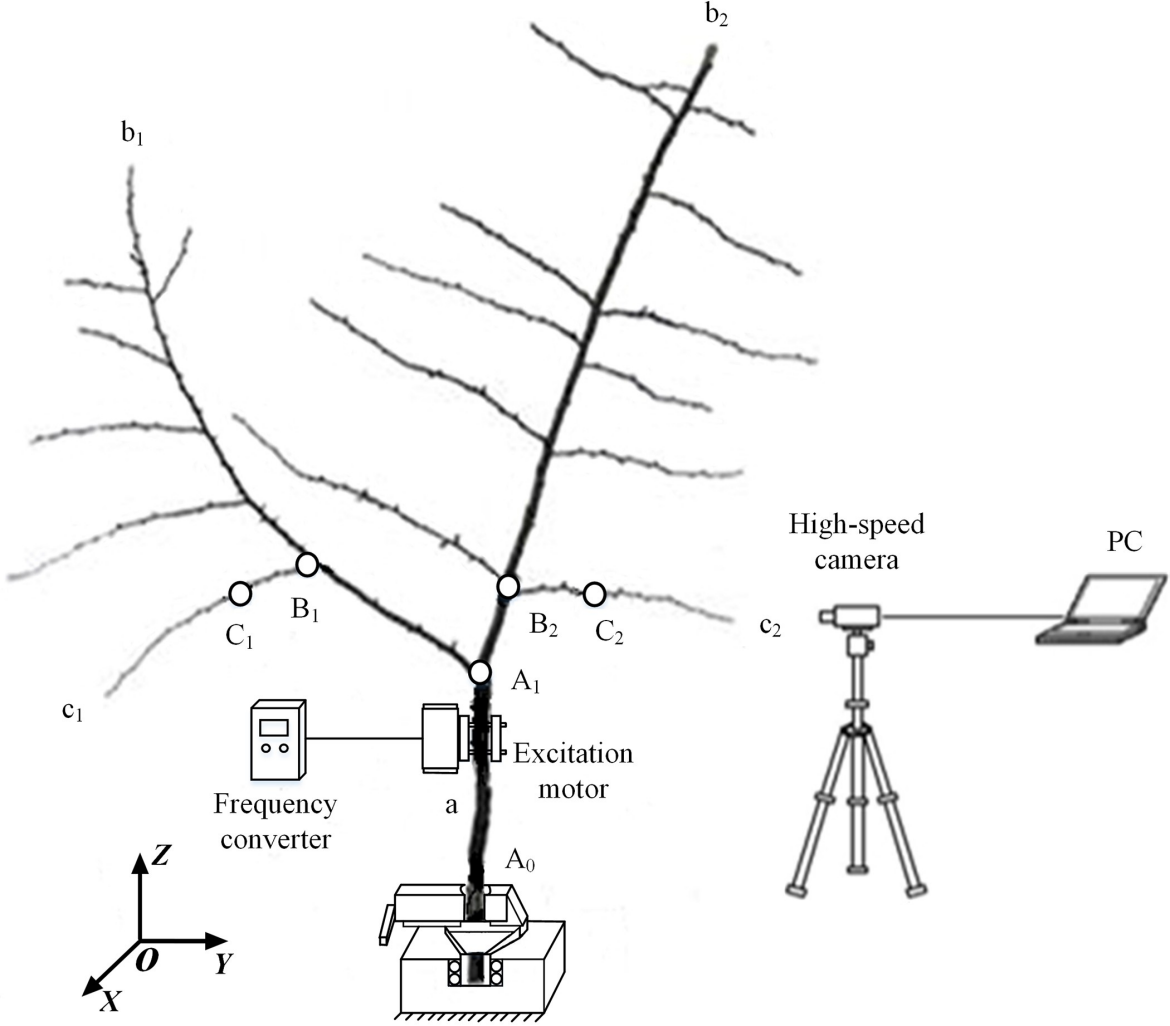


The shape of a fruit tree can be divided into the following categories: sympodial branching, single Y-shaped sympodial branching, and multi-stage Y-shaped sympodial branching. In this paper, a single small fresh Y-shaped sympodial branching Ginkgo biloba tree was selected as the study material from Nanjing Forestry University (China, Jiangsu, Nanjing, 32.1^。^N, 118.8^。^E). The roots of the tree were removed and then the lower end of the tree was fixed in a ground clamp in the laboratory (Fig 1). The trunk “a” denotes segment A_0_A_1_; here the branch began to bifurcate from the trunk A_1_ to form the primary branch b_1_, which meant that the trunk “a” grew at an angle to the other side, named as the primary branch b_2_. Then a branch shape with a ‘Y’-shaped characteristic was formed. The secondary branches c_1_ and c_2_ appeared at the points B_1_ and B_2_ on the primary branches b_1_ and b_2_, and two test points C_1_ and C_2_ were selected by the authors. The height of the Ginkgo biloba tree’s trunk segment A_0_A_1_ was 86.3 cm, and the length of the branches b_1_ and b_2_ were 173.8 cm and 176.6 cm respectively, the rest of the specific characteristic parameters had been shown in Table 1.

**Table 1:** The main parameters of the Ginkgo biloba tree.

**Position**	**A**_**0**_	**A**_**1**_	**B**_**1**_	**C**_**1**_	**B**_**2**_	**C**_**2**_
Diameter/cm	4.15	3.57	1.62	0.54	2.61	0.63
**Segment**	**A**_**0**_**A**_**1**_	**A**_**1**_**B**_**1**_	**A**_**1**_**B**_**2**_	**B**_**1**_**C**_**1**_	**B**_**2**_**C**_**2**_	
Length/cm	74.8	55.2	23.8	20.7	16.1	

### 2.2 The production process of the simulated Ginkgo biloba fruit and verifying its feasibility

The work in this paper has mainly studied the different movement response laws of the Ginkgo biloba fruit at different excitation frequencies, especially at the resonant frequency of the Ginkgo biloba tree; this means that multiple repeated tests were performed at the same position of the fruit. The weight and the fruit’s pedicel retention force of the Ginkgo biloba fruit constantly changes during its different growth stages. When the repeated vibration test is performed with real Ginkgo biloba fruit, even during the same growth period or a short growth time, not only can the fruit and the fruit stalk be easily separated but it can also fall off which means that the test cannot be continued. Moreover, even if the fruit does not fall off, due to the natural physical properties of the fruit stalk constantly changing with the increase of the number of vibrations, the movement response patterns formed by the fruit can also change. Therefore, this makes it difficult to maintain the consistency of repeated tests and the fruit’s movement response changes with the response results when performing tests on the change of the excitation parameters or the scientific nature of the parameters; that is, the use of real Ginkgo biloba fruit cannot meet the test conditions of this paper. Therefore, simulated fruit was used in this paper instead of real Ginkgo biloba fruit in order to achieve consistent results.

**Fig 2:**
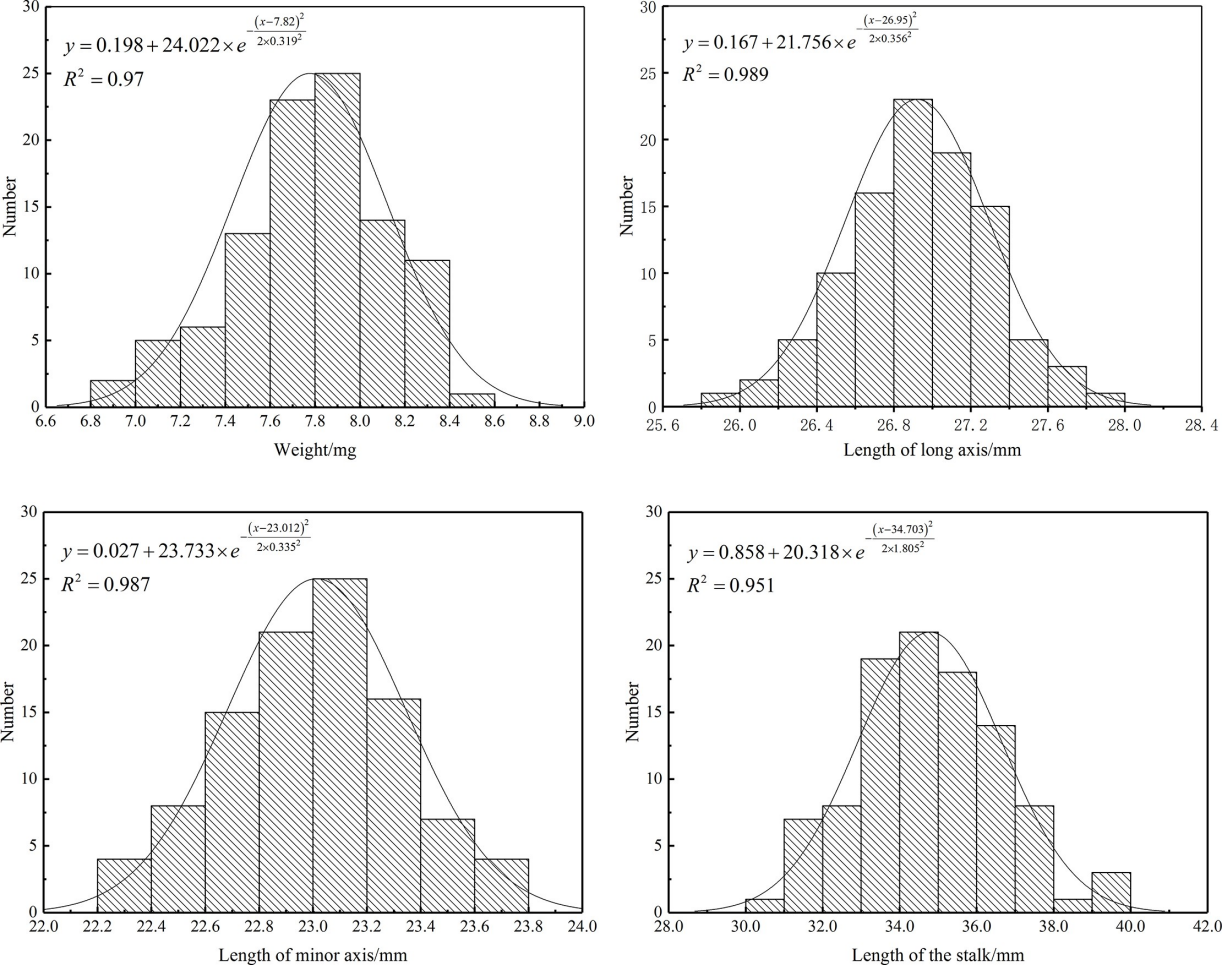


The question of whether simulated Ginkgo biloba fruit can represent the Ginkgo biloba fruit on an actual fruit tree can be verified by comparative experiments. The average parameter values of the fruit were obtained by measuring weight, length of the stalk, length of the long axis and length of the minor axis (Supposing the fruit was a revolving body with an ellipse cross section, the long axis was the long axis of the ellipse, and the minor axis was the short axis of the ellipse) of 100 Ginkgo biloba fruits in their mature phase (mid-September) on four Ginkgo biloba trees in the field. The weight, length of the stalk, length of the long axis and length of the minor axis of fruits were shown in Fig 2. The distributions in figure were fitted by Gaussian curve, respectively.

The weight, length of the stalk, length of the long axis and length of the minor axis of the simulated fruit were determined by the mean of the four distributions above. A simulated fruit with a long axis of 27 mm, a minor axis of 23 mm, a stalk length of 35 mm, and a weight of 7.8 g was re-made by the method of 3D printing. An elastomers line was used to simulate the fruit’s stalk to connect the simulated fruit to the branch. The simulated fruit was then used to carry out the following experiments. A Ginkgo biloba tree with many mature Ginkgo biloba fruit was selected from the Nanjing Forestry University, the test date was the 25th of September, 2018. A Ginkgo biloba fruit on one of the tree’s branches was taken as the observation object. An eccentric-block type excitation motor was used to excite the fruit tree, and a high speed camera (M310, Vision Research Inc, USA) was used to record the vibrational response of the fruit. The fruit was then removed from the branch, and then the simulated fruit was attached to the same position. The fruit tree was then excited at the same excitation frequency and vibration time was 5s, the vibrational response of the simulated fruit was recorded. The maximum displacement of the fruit at different frequencies were shown in Table 2.

**Table 2:** The maximum displacement of the fruit at different frequencies.

Frequency/Hz	10	15	20	25
Real fruit displacement/mm	24.8	40.3	16.3	8.3
Simulated fruit displacement/mm	27.3	45.6	18.2	9.5

**Fig 3:**
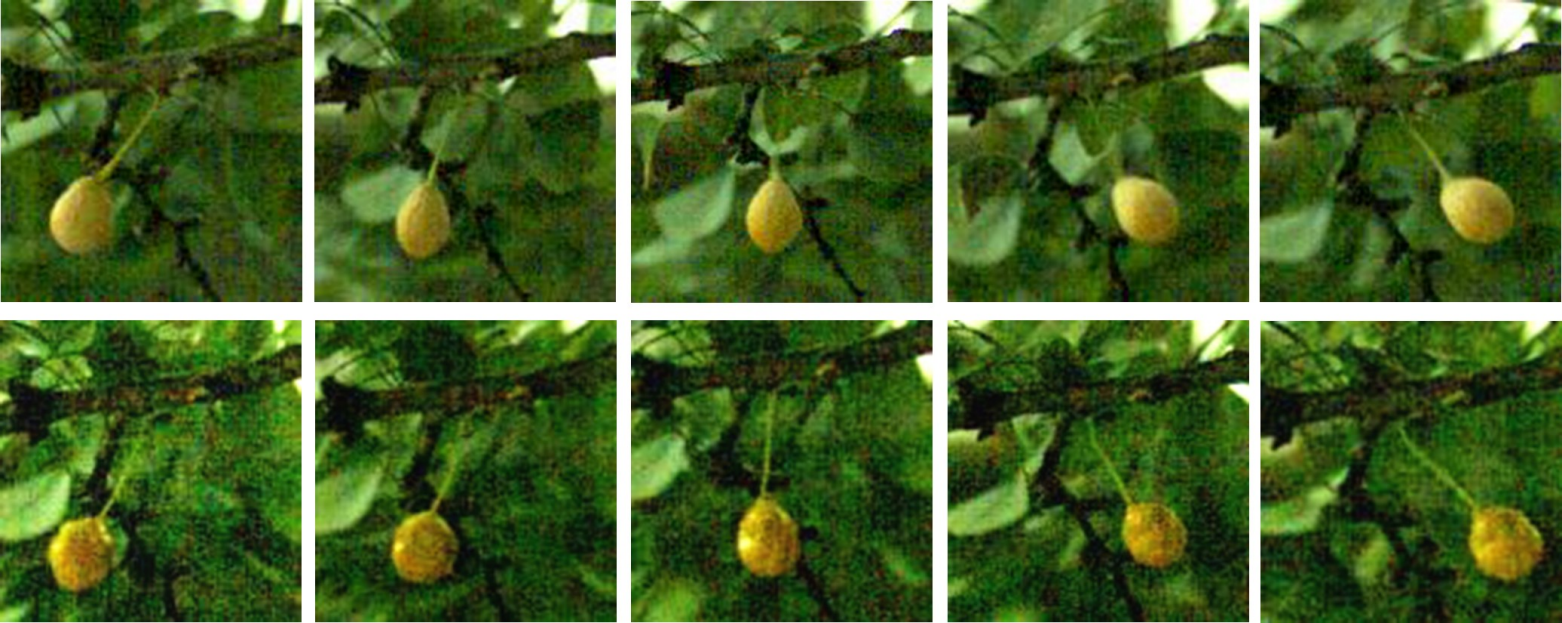


From the Table 2, it was clear that the displacement of the real fruit and the simulated fruit was similar based on small displacement error. At 15 Hz, the vibrational responses of the real fruit and the simulated fruit in a half cycle, according to the same response time, were measured and then a comparative analysis of the vibration position and the vibrational response of the fruit were performed (as shown in Fig 3). The simulated fruit and the real fruit were circular arc-shaped periodic movements, and the trajectory was similar. In addition, comparison tests at 10Hz、15Hz、20Hz and 25Hz were carried out. The results at these frequencies had shown that the simulated fruit could be used to replace the real fruit in order to perform a series of tests to measure the excitation and response characteristics of the fruit.

**Fig 4:**
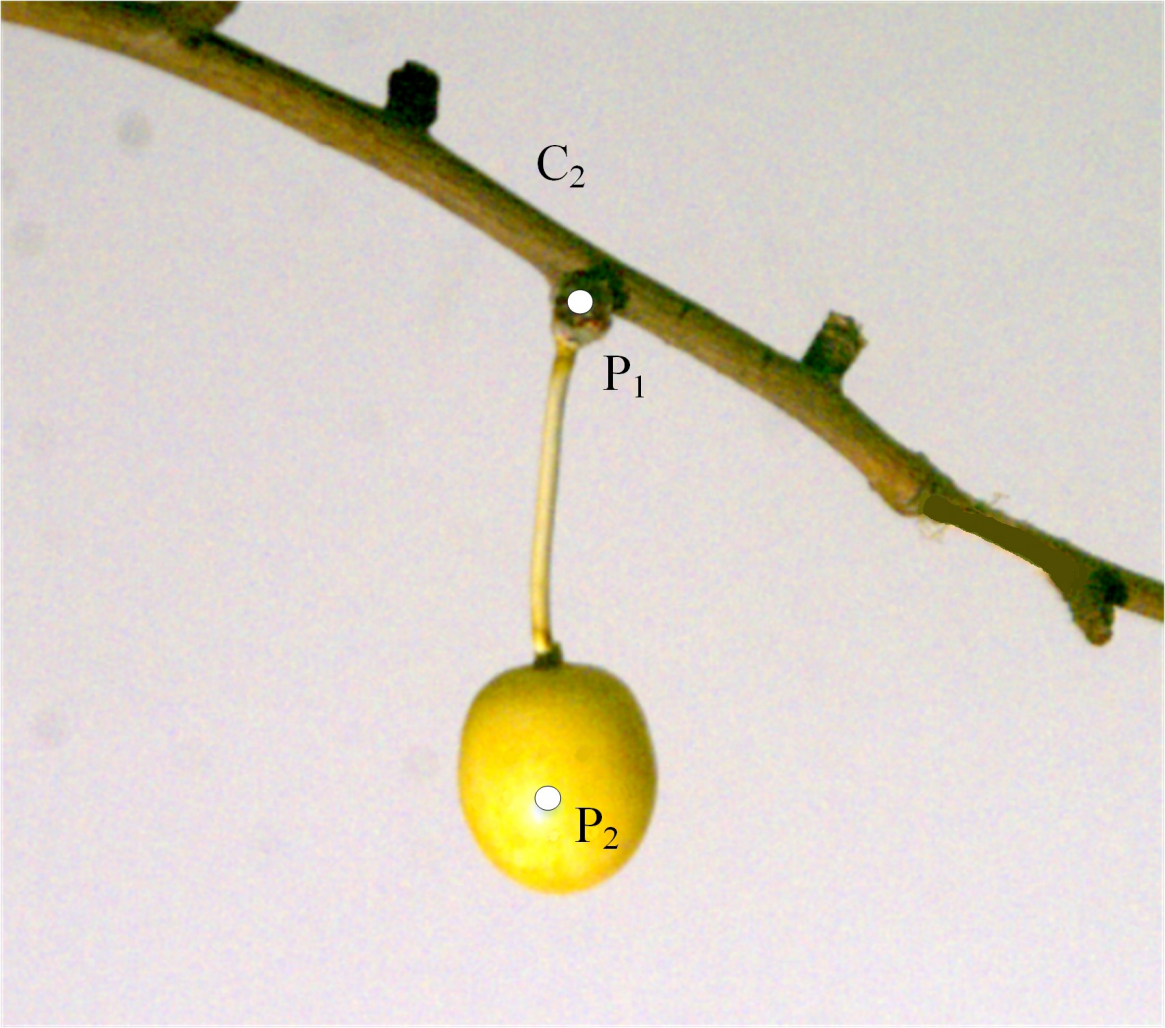


Fig 4 had shown the schematic diagram of the simulated fruit attached to point C_2_ on the sample tree. In order to observe the movement pattern of the fruit under different excitation conditions, the point where the fruit’s stalk joined the fruit branch was marked as P_1_ and the point P_2_ was set at the central position of the fruit. The TEMA image analysis software of the high-speed camera was used to capture the movement trajectories of these two points.

### 2.3 Test equipment and methods

The tree’s frequency spectrum characteristics were tested by using the hammering pulse method, the trunk was struck by an impact hammer, and then the acceleration response of the test point was obtained using a triple-axis accelerometer. The tree’s frequency spectrum characteristics were obtained through frequency spectrum analysis of the acceleration information. The test devices included an impact hammer (LC-02A, Jiangsu Sinocera Piezotronics Inc, Yangzhou, China), four triple-axis accelerometers (CA-YD-141, Jiangsu Sinocera Piezotronics Inc, Yangzhou, China), a charge amplifier (YE5853A, Jiangsu Sinocera Piezotronics Inc, Yangzhou, China), a data acquisition unit (NI cDAQ-9174, the National Instrument Co., Ltd, USA), and analysis software (CRAS V7.1, The First Test Software Engineering, Co., Ltd, Nanjing, China). The triple-axis accelerometer was attached to a small wooden block (15mm×15mm×15mm) by screws, and the small wooden block and the branch’s test points were attached using hot melting adhesive. The point of the trunk of Ginkgo biloba that was hit by the impact hammer was located 60 cm from the lower end of its trunk (A_0_).

For the process of the specific frequency excitation and response, the acceleration response of each test point was measured by clamping the excitation motor to the trunk and then causing excitation of the tree at a specific frequency. The test devices included an excitation device, an acceleration response test device, and an image acquisition device (as shown in Fig 1).

The excitation device consisted of a single-eccentric type excitation motor (Puta Vibrating Motor, Xin Jia Hong Technology Co., Ltd. Shenzhen, China) and a frequency converter (JVFT-S5, Jinhui Instrumentation, Co., Ltd. Shenzhen, China). The excitation motor was fixed to the trunk of the Ginkgo biloba tree using a clamping device and was then connected to the frequency converter, at a distance of 50 cm from the lower end of the trunk (A_0_).

A high speed camera (M310, Vision Research Inc, USA) was used as the image acquisition device, in order to record the fruit’s dynamic response images at a sampling frequency of 1000 fps.

The acceleration response test device that was used was the same as the acceleration test equipment that was used in the tree’s frequency spectrum test method.

The coordinate system *XYZ* was defined as follows: the direction of the trunk growth was defined as the *Z* direction. The line connecting the center of rotation of the eccentric block and the center of the trunk in the horizontal direction was defined as the *X* direction. The *Y* direction was defined as the horizontal direction that was perpendicular to both the *X* and *Z* directions (as shown in Fig 1). The *XYZ* installation direction of each test point of the triple-axis accelerometer on the Ginkgo biloba tree was consistent with the direction set by the *XYZ* coordinate system.

### 2.4 Method of processing the dynamic images of the fruit

The series of images were taken by the image acquisition system could only reflect the individual independent positions of the fruit at different times. In order to reflect the overall dynamic displacement state of the fruit in one cycle, Photoshop software was used to perform translucent processing on the moving images of a single fruit and splice them into a dynamic characteristic image that contained multiple movement positions of the fruit by superimposing multiple images onto one photo. At the same time, through dimensional calibration, MATLAB was used to calculate the displacement of each test point in both the *X* and *Z* directions.

### 2.5 Theoretical analysis of the fruit’s vibrational response

Assuming that the fruit tree is excited at the frequency *ω*, and the fruit on a certain fruit branch responds at the same frequency *ω*, then the vibrational displacement response of the fruit in the *X*, *Y* and *Z* directions can be written as:

$$\{\begin{array}{c} x(t)=A_{x}\sin(\omega t)\\ y(t)=A_{y}\sin(\omega t)\\ z(t)=A_{z}\sin(\omega t) \end{array},$$ (1)

Where, *Ax*, *Ay*, and *Az* correspond to the vibration amplitudes of the *x*, *y*, and *z* directions, respectively.

The corresponding acceleration response (regardless of the initial phase) could be written as:

$$\{\begin{array}{c} a_{x}(t)=-A_{x}\omega^{2}\sin(\omega t)\\ a_{y}(t)=-A_{y}\omega^{2}\sin(\omega t)\\ a_{z}(t)=-A_{z}\omega^{2}\sin(\omega t) \end{array},$$ (2)

As a result, there is a *ω*^2^-rate relationship between the acceleration amplitude *ω*^2^ and the displacement amplitude *A*.

## 3. Results and discussion

### 3.1 Frequency spectrum characteristics of the Ginkgo biloba fruit tree

**Fig 5:**
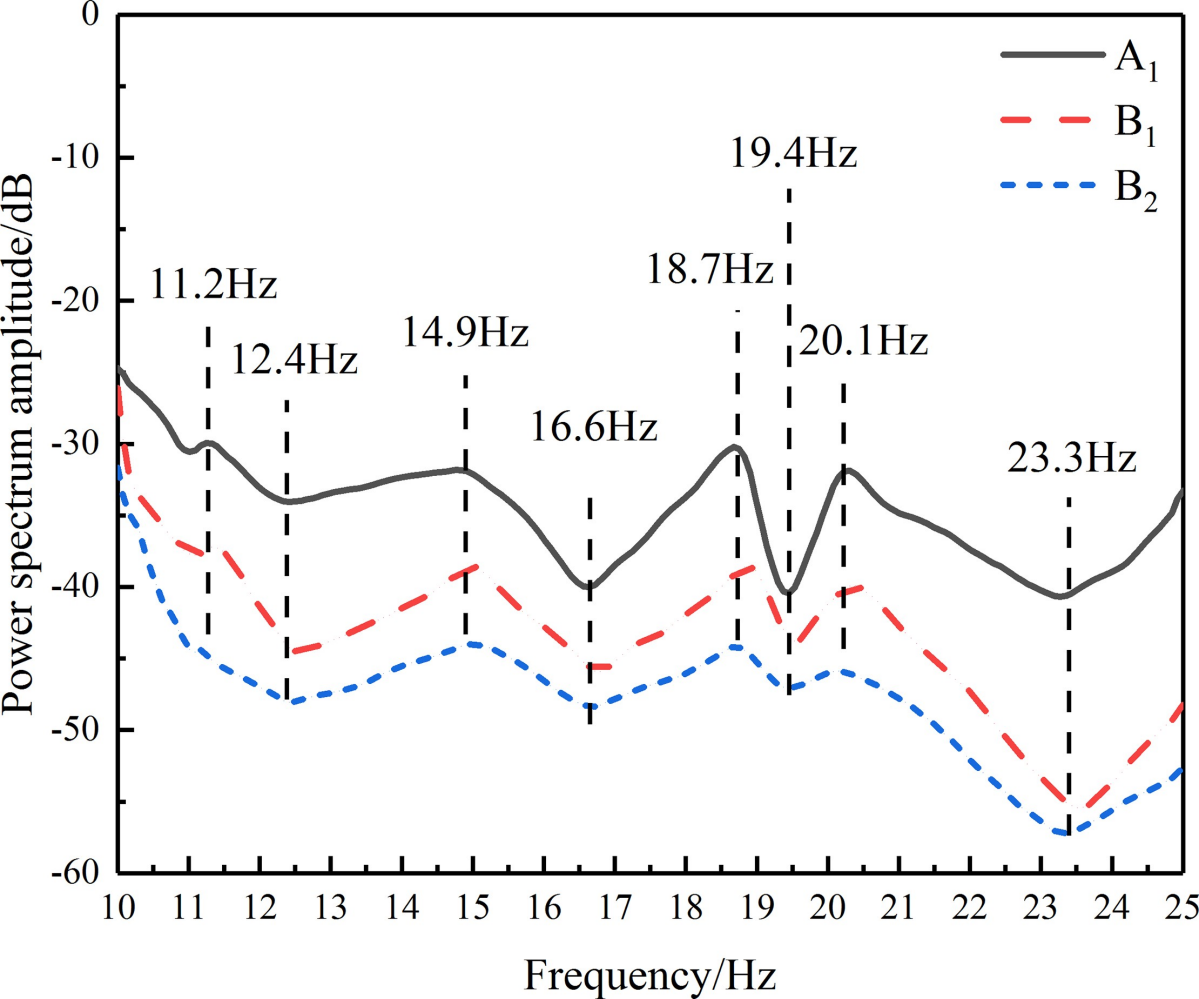


The vibrational response of each fruit bearing branch of the fruit tree was caused by excitation of the tree trunk. The small displacement vibration demonstrated by each branch of the fruit tree could be considered as linear vibration of the tree. In order to study the resonant response caused by harmonic excitation of the fruit tree, the frequency spectrum curve of the fruit tree needed to be determined. The tree trunk was struck by the hammer in *X* direction, which introduced the impact signals into the fruit tree, and the triple-axis accelerometer was installed on the test point A_1,_ B_1_ and B_2_ on the trunk. Tapping the tree in *X* direction, the peak of the spectrum curve in *X* direction was higher and more clearly reflects the natural frequency, because the energy in tapping direction was bigger than others. So the curve in *X* direction was used to select the natural frequency. The spectral characteristic curves of three points in *X* direction were obtained as shown in Fig 5. It was found that the frequency spectrum curves had great consistency in three points [28].

In general, the range of excitation frequency used in fruit harvesting, based on the eccentric block type excitation method, does not exceed 25 Hz, because when the excitation frequency exceed 25 Hz, the probability of damaging fruit trees was very high. Therefore, this paper focused on the frequency spectrum curves below 25 Hz. Through excitation of a plurality of harmonic frequency points in the low frequency region (0~10 Hz), an effective vibrational response of the fruit tree cannot be induced, so the frequency spectrum curve range that was used in this paper was reduced to the range 10~25 Hz. The frequencies that corresponded to the peak points on the curve in this frequency range might be the resonant frequencies that easily caused resonance of the fruit tree, the frequencies were 11.2 Hz, 14.9 Hz, 18.7 Hz, and 20.1 Hz. The frequencies 12.4 Hz, 16.6 Hz, 19.4 Hz, and 23.3 Hz, corresponding to the trough, were treated as the contrast frequencies of the fruit trees’ excitation and response. The trunk of the fruit tree was excited at these above characteristic frequencies in order to determine the fruit’s vibrational response at the four test points B_1_, B_2_, C_1_ and C_2_.

### 3.2 Acceleration response of the test points

The secondary branches of the tree c_1_ and c_2_ were very soft; therefore if the accelerometer had been installed at the test points C_1_ and C_2_, it would have a large loading effect on the response of the branch. The resonant frequencies of B_1_ and B_2_ were very similar to the resonant frequencies of points C_1_ and C_2_, respectively. Therefore, the acceleration response at different characteristic frequencies was only tested at the branching points B_1_ and B_2_ of the secondary branches. During the acceleration response experiments, there was only one simulated fruit at the location of the test point B_1_ and B_2_. The results of the test have been shown in Table 3.

**Table 3:** The acceleration amplitude values of test points B_1_ and B_2_ (m/s^2^).

Frequency/Hz	Test point B_1_				Test point B_2_			
	*X* direction	*Y* direction	*Z* direction	resultant acceleration	*X* direction	*Y* direction	*Z* direction	resultant acceleration
11.2	28.44	18.27	5.51	34.25	27.18	7.08	4.91	28.51
12.4	15.16	11.35	2.18	19.06	12.64	4.02	4.43	13.98
14.9	26.26	25.19	6.65	36.99	26.19	4.60	5.43	27.14
16.6	16.04	16.15	2.94	22.95	14.68	3.73	4.24	15.73
18.7	35.81	28.91	8.98	46.89	28.97	7.21	7.16	30.70
19.4	25.21	12.60	7.45	29.15	15.23	4.78	4.34	16.54
20.1	55.90	36.51	28.76	72.70	40.39	9.48	7.57	42.17
23.3	56.31	31.19	15.65	66.25	19.46	8.95	5.42	22.09

**Fig 6:**
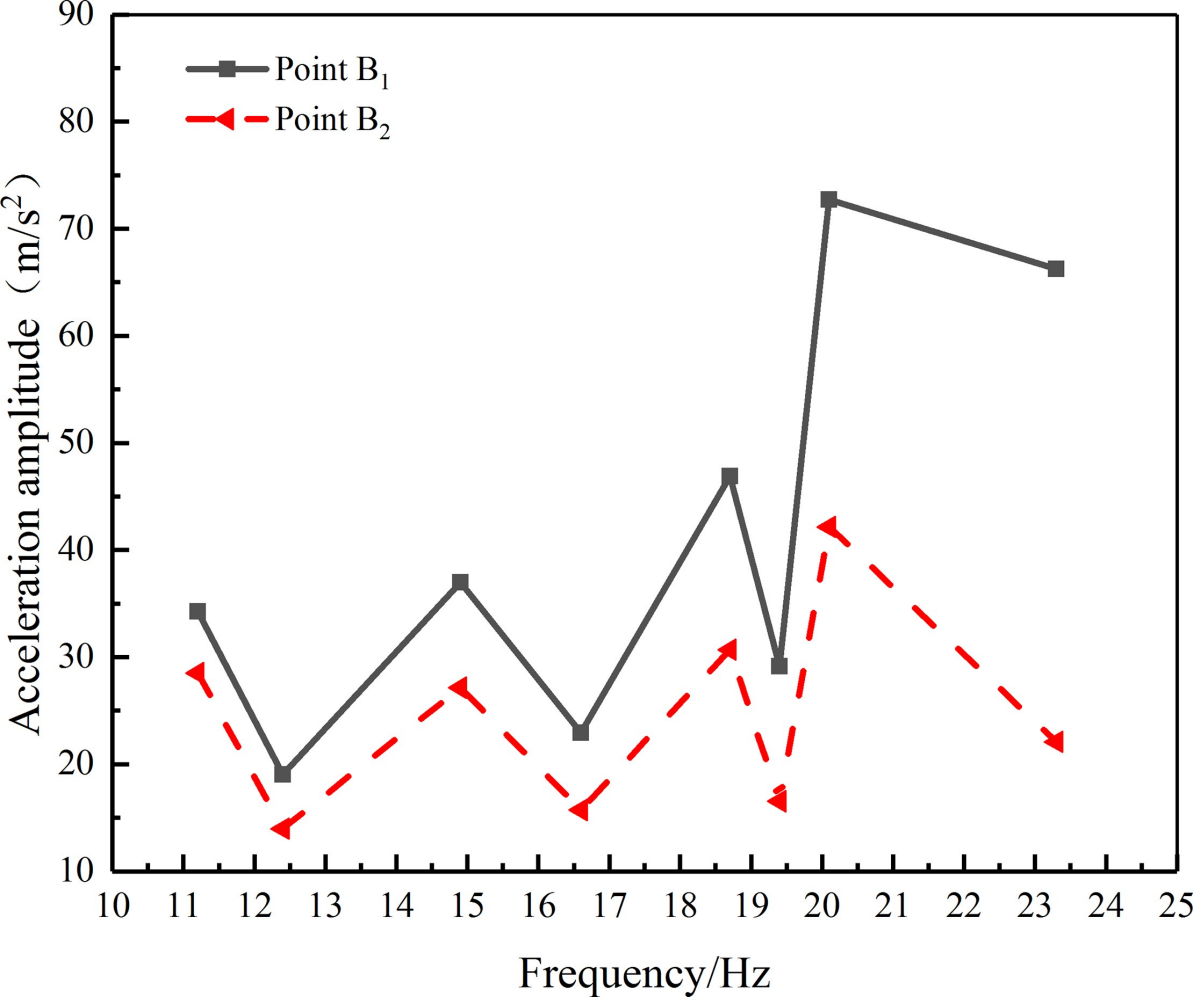


For test point B_1_, the acceleration response values of the *X*, *Y*, and *Z* directions were different for each frequency. The acceleration response value in the *X* direction was the largest, the value in the *Y* direction was larger except at 16.6 Hz, and the value in the *Z* direction was much smaller than those of both the *X* and *Y* directions. For example, when the excitation frequency was 11.2 Hz, the acceleration value in the *Z* direction was only 5.51 m/s^2^, while the acceleration values in the *X* direction and *Y* direction were 5.16 times and 3.32 times of that in the *Z* direction respectively. The acceleration response value in the *X* direction was larger, which was related to the connection between the center of rotation of the exciter’s eccentric block and the center of the trunk being set in the *X* direction. At the same time, under the action of the excitation force, the amplitude of the lateral swing or shaking of the trunk was very small, which resulted in the vibration amplitude or acceleration amplitude in the *Z* direction at test point B_1_ being the smallest. When the excitation frequency matched the resonant frequency that corresponded to the peak in the spectrum shown in Fig 5, the corresponding harmonic response appeared, and the corresponding acceleration amplitude was significantly higher than the response caused by the trough frequency excitation. It could be seen from the frequency and acceleration response (shown in Fig 6) that the excitation at the resonant frequency caused a strong harmonic response.

For test point B_2_, the overall response law was basically consistent with that of test point B_1_. Excitation at the resonant frequency also caused a strong harmonic response, however, the acceleration amplitudes in the *Y* and *Z* directions were much smaller than that in the *X* direction and the difference between the two was small, which meant that the resultant acceleration value, especially the resultant acceleration value that corresponded to the harmonic response was significantly lower than that of test point B_1_.

### 3.3 Movement response trajectory and the movement patterns of fruit

**Fig 7:**
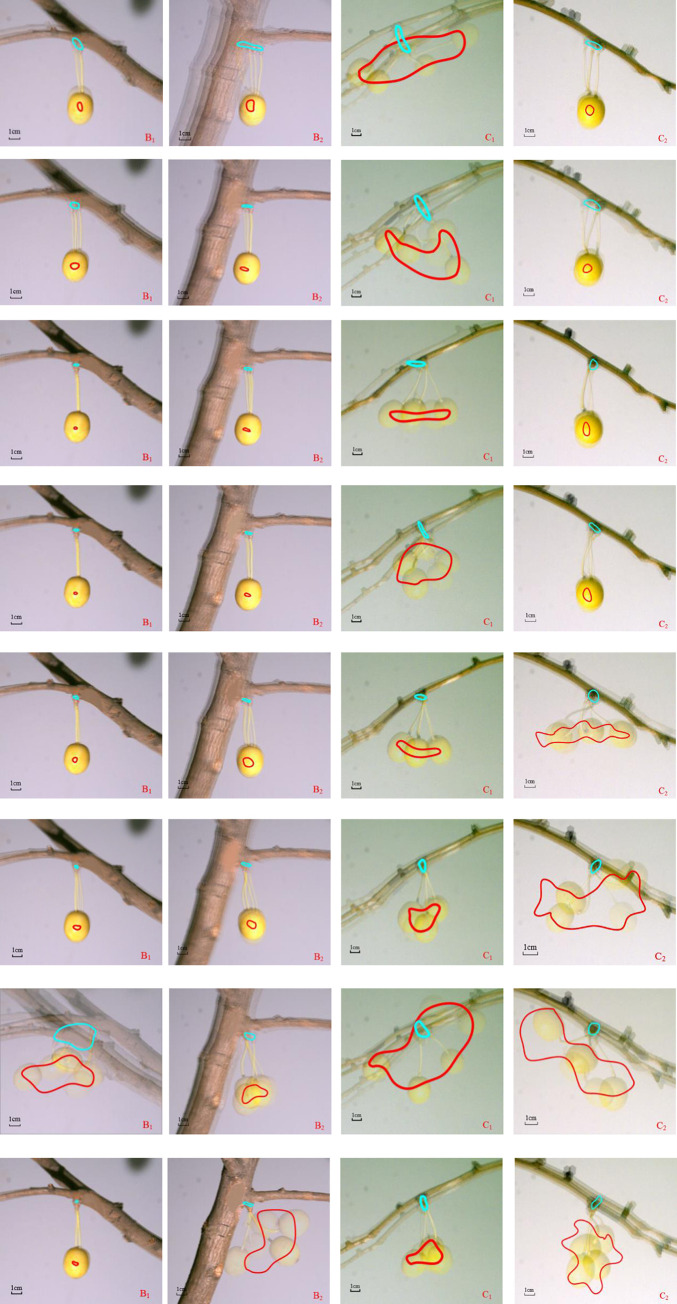


The vibrational response of the fruit was induced by a cycle caused by excitation at each frequency. By tracking the join of the fruit stalk and the fruit branch and the join of the fruit stalk and the fruit (points P_1_ and P_2_ in Fig 4), the respective movement trajectory diagram could be formed, as shown in Fig 7, in which each small figure shows the overlapping effect graph of each fruit in five typical positions within one cycle at the corresponding excitation frequency.

When the trunk was excited at a resonant frequency of 11.2 Hz (Fig 7A), the point P_1_ of test point B_1_ formed an irregular small oblique ellipse (corresponding to a maximum horizontal displacement of 8.6 mm and a vertical displacement of 9.4 mm), and the response of point P_2_ on the fruit was smaller (horizontal displacement of 4.3 mm and vertical displacement of 7.6 mm). The branch c_1_ formed by the bifurcation of point B_1_, under the weak vibration of B_1_, produced a vibration mode that mainly oscillated in the vertical direction. The horizontal displacement of the point P_1_ at test point C_1_ on branch c_1_ was smaller, while point P_1_ was subjected to a larger displacement of 27.5 mm in the vertical direction, which caused the fruit to produce a very strong complex movement with large displacement, oscillation and beating. The maximum horizontal and vertical displacement corresponding to the trajectory of point P_2_ was 104.2 mm and 56.5 mm, respectively. It was only because the offshoot b_1_ was bifurcated, which caused the main trunk “a” to be obviously deflected to form the offshoot b_2_, that the offshoot b_2_ was basically an extension of the trunk “a”. Therefore, when excitation of the trunk “a” caused the trunk to oscillate, test point B_2_ on the offshoot b_2_ showed obvious oscillation in the horizontal direction, and the maximum horizontal displacement corresponding to point P_1_ reached 22.7 mm during the oscillation. However, the point P_2_ on the fruit did not cause an obvious movement response. The offshoot c_2_ from the bifurcation of point B_2_ was also dominated by the horizontal pulling movement, and the fruit’s movement response at the test point C_2_ on this branch was also weaker.

When the trunk was excited by a trough point frequency of 12.4 Hz (Fig 7B), the movement response of the fruit at the test points B_1_, B_2_, and C_2_ was not very different from that caused by an excitation frequency of 11.2 Hz. However, due to the trajectory of point P_1_, the vertical trajectory was dominant at test point C_1_, and its vertical displacement reached 22.3 mm, which caused the fruit to produce a strong movement form mainly with beating and oscillation.

When excited at a resonant frequency of 14.9 Hz (Fig 7C) and a trough frequency of 16.6 Hz (Fig 7D), the vibrational responses of the fruit at the test points B_1_, B_2_ and C_2_ were all very weak. The response of the fruit at point C_1_ was relatively obvious, but not as strong as the first two frequencies. Corresponding to a frequency of 14.9 Hz, the movement trajectory of the point P_1_ at point C_1_ mainly involved horizontal movement, and the fruit mainly moved in the form of oscillating movement. Corresponding to a frequency of 16.6 Hz, the movement trajectory of the point P_1_ at point C_1_ mainly involved vertical movement, and the fruit mainly displayed a complex movement form with beating and oscillation.

When excited at a resonant frequency of 18.7 Hz (Fig 7E) and a trough frequency of 19.4 Hz (Fig 7F), the vibrational responses of the fruit at the test points B_1_ and B_2_ were very weak. The movement trajectories and movement forms of the points P_1_ and P_2_, corresponding to the fruit at point C_1_, were very similar to those at the excitation frequencies of 14.9 Hz and 16.6 Hz, although the amplitudes of the responses were smaller. The maximum horizontal displacement of the point P_2_ also reached 42.9 mm and 30.4 mm. However, the fruit at point C_2_ had a strong response at a frequency of 18.7 Hz. The response caused by a trough frequency of 19.4 Hz was stronger than the fruit’s response to the resonant frequency of 18.7 Hz. The maximum horizontal and vertical displacement that corresponded to the movement trajectory of the point P_2_ on the fruit was 81.4 mm and 41.9 mm, and the movement form of the fruit was mainly composed of a combination of beating, flipping, and oscillation.

When excited at the resonant frequency of 20.1 Hz (Fig 7G), not only did the fruit at the points C_1_ and C_2_ on the secondary branches produce very strong vibrational responses, but also all the fruit at the points B_1_ and B_2_ produced obvious vibrational responses for the premise that all frequencies that were lower than this frequency failed to induce vibrational responses in the fruit at points B_1_ and B_2_ on the first-rank branch. However, the fruit’s response at point B_2_ was not as strong as that of point B_1_. When excited at a trough point frequency of 23.3 Hz (Fig 7H), the response of the fruit at point B_1_ was negligible. At this frequency, the fruit’s response at point B_2_ was very strong. The fruit at points C_1_ and C_2_ had a certain degree of vibrational response, but it was far less intense than that at 20.1 Hz.

## 4. Conclusions

From the comparison tests between the simulated fruit and the real Ginkgo biloba fruit, the results showed that the simulated fruit and the Ginkgo biloba fruit had better frequency response consistency, and that the simulated fruit can be used to replace the real Ginkgo biloba fruit in order to perform a large number of repeatable tests.

The frequency curve of the fruit tree that was obtained was based on the pulse hammering method. From the results, four resonant frequencies and four trough point frequencies in the frequency range of 10Hz~25Hz were determined as the experimental excitation frequency of the Ginkgo biloba fruit in this paper. The fruit’s acceleration responses to the resonant frequency and the trough point frequency at two test points on the two primary branches on the Ginkgo biloba tree were analyzed. The resonant frequency caused an obvious harmonic response from the fruit.

The vibrational response of the fruit at the different points on the fruit tree was not consistent. At the same frequency, fruit at certain points produced a very strong vibrational response, while at other points, the fruit was almost stationary. Therefore, it was difficult for a fruit tree to completely shed all its fruit through the excitation method at a single frequency unless the excitation amplitude was very large. Therefore the excitation method should cover a range of frequencies to achieve maximum fruit shedding.

The primary branches of the tree that had a greater stiffness made it more difficult to induce a strong response from the fruit. Conversely, the softer secondary branches made it easy to induce strong vibrational response from the fruit regardless of the resonant frequency or the trough point frequency.

## Supporting information

S1 Data (DOCX)
