# Supplementary material for: The vibrational response of simulated Ginkgo biloba fruit based on their frequency spectrum characteristics
Source: PLoS One. 2020 Jul 23;15(7):e0235494. doi: 10.1371/journal.pone.0235494 (PMC7377492; doi:10.1371/journal.pone.0235494)
Supplement: S1 Data — (DOCX) [file pone.0235494.s001.docx]

# Minimal Data Set

**Table 1. The main parameters of the Ginkgo fruit.**

| **Number** | **Weight/mg** | **Length of the stalk/mm** | **Length of long axis/mm** | **Length of minor axis/mm** |
| --- | --- | --- | --- | --- |
| 1 | 7.58 | 37.4 | 26.9 | 22.7 |
| 2 | 7.26 | 36.5 | 27.0 | 23.0 |
| 3 | 8.09 | 36.0 | 27.0 | 22.9 |
| 4 | 7.43 | 36.6 | 26.4 | 22.9 |
| 5 | 8.21 | 34.9 | 26.8 | 22.7 |
| 6 | 7.66 | 34.9 | 26.9 | 23.0 |
| 7 | 7.68 | 37.0 | 26.5 | 23.2 |
| 8 | 8.32 | 37.2 | 26.4 | 22.7 |
| 9 | 7.90 | 34.1 | 26.9 | 22.5 |
| 10 | 7.64 | 31.4 | 27.1 | 22.8 |
| 11 | 7.47 | 33.4 | 27.7 | 23.3 |
| 12 | 7.59 | 31.8 | 27.3 | 22.5 |
| 13 | 7.96 | 33.0 | 26.4 | 22.7 |
| 14 | 8.14 | 35.9 | 26.9 | 23.4 |
| 15 | 7.82 | 33.4 | 27.1 | 22.8 |
| 16 | 7.60 | 34.8 | 26.9 | 23.5 |
| 17 | 7.93 | 35.7 | 27.3 | 23.2 |
| 18 | 8.07 | 32.2 | 27.5 | 23.3 |
| 19 | 7.63 | 33.3 | 26.9 | 23.0 |
| 20 | 8.38 | 33.5 | 27.1 | 22.9 |
| 21 | 7.65 | 33.4 | 26.6 | 22.9 |
| 22 | 8.16 | 34.1 | 27.6 | 23.1 |
| 23 | 8.19 | 36.6 | 27.0 | 23.4 |
| 24 | 7.82 | 34.0 | 26.7 | 23.4 |
| 25 | 7.77 | 36.4 | 26.9 | 23.0 |
| 26 | 8.15 | 35.7 | 26.7 | 23.3 |
| 27 | 7.08 | 35.4 | 27.3 | 23.1 |
| 28 | 7.81 | 37.0 | 27.2 | 22.8 |
| 29 | 7.83 | 39.3 | 27.2 | 23.1 |
| 30 | 7.95 | 37.9 | 27.2 | 22.7 |
| 31 | 7.45 | 31.8 | 27.2 | 23.3 |
| 32 | 7.24 | 36.1 | 27.0 | 23.5 |
| 33 | 7.34 | 32.5 | 27.9 | 23.5 |
| 34 | 8.02 | 31.0 | 26.9 | 23.1 |
| 35 | 8.23 | 32.6 | 26.6 | 22.3 |
| 36 | 7.62 | 36.6 | 26.8 | 22.9 |
| 37 | 7.75 | 32.7 | 27.6 | 23.2 |
| 38 | 7.54 | 37.7 | 27.4 | 23.0 |
| 39 | 7.28 | 34.0 | 27.1 | 23.7 |
| 40 | 7.74 | 39.4 | 26.9 | 23.3 |
| 41 | 7.84 | 35.8 | 27.1 | 22.7 |
| 42 | 8.07 | 37.0 | 26.7 | 23.3 |
| 43 | 7.85 | 34.7 | 26.9 | 23.3 |
| 44 | 7.49 | 35.4 | 26.9 | 23.6 |
| 45 | 7.19 | 35.4 | 27.0 | 23.0 |
| 46 | 7.57 | 35.3 | 26.7 | 23.1 |
| 47 | 7.25 | 32.0 | 26.9 | 23.2 |
| 48 | 8.09 | 33.3 | 27.2 | 22.8 |
| 49 | 7.76 | 32.9 | 26.6 | 22.8 |
| 50 | 7.06 | 35.3 | 26.7 | 22.9 |
| 51 | 7.95 | 38.0 | 27.3 | 22.8 |
| 52 | 7.43 | 39.3 | 26.9 | 22.4 |
| 53 | 8.34 | 31.5 | 26.2 | 23.0 |
| 54 | 7.82 | 35.7 | 26.7 | 22.6 |
| 55 | 7.89 | 33.7 | 26.5 | 23.6 |
| 56 | 7.49 | 32.8 | 27.2 | 23.1 |
| 57 | 8.01 | 35.2 | 27.1 | 23.1 |
| 58 | 7.83 | 34.1 | 26.8 | 23.1 |
| 59 | 7.89 | 35.1 | 26.1 | 22.5 |
| 60 | 7.67 | 36.5 | 27.1 | 23.7 |
| 61 | 8.06 | 33.1 | 27.1 | 22.7 |
| 62 | 7.32 | 34.7 | 26.5 | 23.2 |
| 63 | 7.87 | 31.6 | 26.3 | 22.9 |
| 64 | 7.82 | 34.2 | 26.9 | 23.2 |
| 65 | 7.82 | 37.0 | 27.4 | 23.0 |
| 66 | 7.63 | 31.5 | 27.3 | 23.1 |
| 67 | 7.56 | 35.8 | 26.8 | 22.5 |
| 68 | 7.87 | 32.3 | 26.2 | 23.1 |
| 69 | 7.57 | 35.4 | 26.7 | 22.9 |
| 70 | 8.26 | 34.1 | 26.4 | 22.9 |
| 71 | 7.99 | 34.4 | 27.2 | 23.1 |
| 72 | 8.35 | 31.8 | 26.7 | 22.9 |
| 73 | 7.78 | 33.2 | 27.1 | 23.2 |
| 74 | 7.78 | 34.6 | 26.9 | 23.4 |
| 75 | 7.93 | 35.1 | 26.2 | 22.2 |
| 76 | 8.38 | 34.6 | 27.0 | 22.3 |
| 77 | 6.84 | 33.7 | 27.4 | 22.7 |
| 78 | 7.13 | 37.7 | 26.8 | 23.2 |
| 79 | 8.31 | 34.6 | 27.3 | 23.2 |
| 80 | 8.36 | 33.4 | 27.2 | 22.8 |
| 81 | 7.72 | 33.5 | 27.2 | 23.3 |
| 82 | 8.34 | 34.8 | 26.8 | 23.0 |
| 83 | 7.67 | 36.9 | 26.9 | 22.5 |
| 84 | 7.88 | 34.2 | 26.9 | 22.6 |
| 85 | 7.62 | 36.7 | 26.9 | 22.9 |
| 86 | 7.61 | 35.5 | 26.3 | 23.1 |
| 87 | 7.70 | 33.9 | 27.0 | 23.4 |
| 88 | 8.12 | 33.4 | 26.4 | 22.8 |
| 89 | 7.73 | 35.4 | 27.1 | 23.5 |
| 90 | 8.41 | 34.8 | 26.4 | 22.9 |
| 91 | 7.66 | 33.2 | 27.7 | 23.1 |
| 92 | 7.51 | 33.4 | 27.4 | 23.5 |
| 93 | 8.02 | 36.1 | 26.7 | 23.3 |
| 94 | 7.89 | 36.0 | 27.0 | 23.2 |
| 95 | 7.99 | 34.4 | 27.6 | 23.7 |
| 96 | 6.96 | 36.7 | 27.4 | 22.5 |
| 97 | 7.74 | 34.3 | 26.0 | 22.8 |
| 98 | 7.07 | 34.8 | 26.4 | 23.1 |
| 99 | 7.87 | 37.5 | 26.6 | 23.0 |
| 100 | 8.11 | 33.9 | 26.8 | 22.7 |

**Table 2. The frequency spectrum characteristics.**

| **Frequency/Hz** | **Point A_1_/dB** | **Point B_1_/dB** | **Point B_2_/dB** |
| --- | --- | --- | --- |
| 10.000 | -24.71 | -26.11 | -31.67 |
| 10.078 | -25.09 | -29.69 | -33.70 |
| 10.156 | -25.80 | -31.97 | -34.88 |
| 10.234 | -26.13 | -33.10 | -35.38 |
| 10.313 | -26.46 | -33.69 | -36.15 |
| 10.391 | -26.82 | -34.19 | -37.65 |
| 10.469 | -27.22 | -34.65 | -38.88 |
| 10.547 | -27.63 | -35.15 | -39.85 |
| 10.625 | -28.12 | -35.69 | -40.92 |
| 10.703 | -28.70 | -36.17 | -41.60 |
| 10.781 | -29.32 | -36.56 | -42.01 |
| 10.859 | -30.00 | -36.97 | -43.05 |
| 10.938 | -30.43 | -37.42 | -43.74 |
| 11.016 | -30.56 | -37.70 | -44.35 |
| 11.094 | -30.46 | -37.78 | -44.57 |
| 11.172 | -30.11 | -37.67 | -44.43 |
| 11.250 | -29.92 | -37.38 | -44.72 |
| 11.328 | -29.96 | -37.23 | -45.10 |
| 11.406 | -30.18 | -37.28 | -45.39 |
| 11.484 | -30.58 | -37.51 | -45.66 |
| 11.563 | -30.95 | -37.93 | -45.86 |
| 11.641 | -31.28 | -38.46 | -46.04 |
| 11.719 | -31.63 | -39.10 | -46.23 |
| 11.797 | -32.03 | -39.77 | -46.44 |
| 11.875 | -32.44 | -40.45 | -46.64 |
| 11.953 | -32.85 | -41.08 | -46.86 |
| 12.031 | -33.20 | -41.63 | -47.06 |
| 12.109 | -33.47 | -42.17 | -47.26 |
| 12.188 | -33.70 | -42.69 | -47.47 |
| 12.266 | -33.89 | -43.25 | -47.72 |
| 12.344 | -34.00 | -43.83 | -47.96 |
| 12.422 | -34.04 | -44.26 | -48.05 |
| 12.500 | -34.03 | -44.49 | -48.00 |
| 12.578 | -33.97 | -44.54 | -47.90 |
| 12.656 | -33.89 | -44.42 | -47.78 |
| 12.734 | -33.80 | -44.28 | -47.68 |
| 12.813 | -33.70 | -44.16 | -47.59 |
| 12.891 | -33.59 | -44.02 | -47.51 |
| 12.969 | -33.48 | -43.86 | -47.43 |
| 13.047 | -33.38 | -43.69 | -47.35 |
| 13.125 | -33.30 | -43.53 | -47.27 |
| 13.203 | -33.24 | -43.36 | -47.19 |
| 13.281 | -33.18 | -43.18 | -47.09 |
| 13.359 | -33.12 | -42.99 | -46.96 |
| 13.438 | -33.05 | -42.80 | -46.80 |
| 13.516 | -32.96 | -42.63 | -46.63 |
| 13.594 | -32.86 | -42.48 | -46.45 |
| 13.672 | -32.74 | -42.32 | -46.26 |
| 13.750 | -32.62 | -42.15 | -46.06 |
| 13.828 | -32.51 | -41.96 | -45.90 |
| 13.906 | -32.42 | -41.76 | -45.73 |
| 13.984 | -32.34 | -41.53 | -45.57 |
| 14.063 | -32.28 | -41.29 | -45.43 |
| 14.141 | -32.23 | -41.08 | -45.30 |
| 14.219 | -32.17 | -40.93 | -45.19 |
| 14.297 | -32.12 | -40.77 | -45.08 |
| 14.375 | -32.07 | -40.59 | -44.97 |
| 14.453 | -32.03 | -40.35 | -44.85 |
| 14.531 | -31.98 | -40.03 | -44.72 |
| 14.609 | -31.93 | -39.72 | -44.58 |
| 14.688 | -31.88 | -39.43 | -44.45 |
| 14.766 | -31.82 | -39.14 | -44.30 |
| 14.844 | -31.83 | -38.86 | -44.15 |
| 14.922 | -31.92 | -38.67 | -44.03 |
| 15.000 | -32.10 | -38.58 | -44.01 |
| 15.078 | -32.36 | -38.68 | -44.05 |
| 15.156 | -32.65 | -38.97 | -44.12 |
| 15.234 | -32.95 | -39.35 | -44.25 |
| 15.313 | -33.24 | -39.78 | -44.46 |
| 15.391 | -33.52 | -40.21 | -44.66 |
| 15.469 | -33.81 | -40.63 | -44.85 |
| 15.547 | -34.14 | -41.02 | -45.05 |
| 15.625 | -34.48 | -41.38 | -45.24 |
| 15.703 | -34.86 | -41.71 | -45.47 |
| 15.781 | -35.27 | -41.99 | -45.73 |
| 15.859 | -35.73 | -42.28 | -46.00 |
| 15.938 | -36.22 | -42.55 | -46.30 |
| 16.016 | -36.75 | -42.86 | -46.60 |
| 16.094 | -37.29 | -43.20 | -46.89 |
| 16.172 | -37.81 | -43.53 | -47.18 |
| 16.250 | -38.34 | -43.84 | -47.46 |
| 16.328 | -38.88 | -44.15 | -47.70 |
| 16.406 | -39.36 | -44.46 | -47.90 |
| 16.484 | -39.75 | -44.83 | -48.08 |
| 16.563 | -39.97 | -45.27 | -48.27 |
| 16.641 | -40.01 | -45.62 | -48.39 |
| 16.719 | -39.86 | -45.87 | -48.36 |
| 16.797 | -39.54 | -45.91 | -48.25 |
| 16.875 | -39.15 | -45.75 | -48.10 |
| 16.953 | -38.72 | -45.47 | -47.93 |
| 17.031 | -38.35 | -45.09 | -47.77 |
| 17.109 | -38.03 | -44.73 | -47.62 |
| 17.188 | -37.74 | -44.39 | -47.46 |
| 17.266 | -37.45 | -44.13 | -47.30 |
| 17.344 | -37.10 | -43.93 | -47.14 |
| 17.422 | -36.67 | -43.77 | -46.99 |
| 17.500 | -36.21 | -43.65 | -46.85 |
| 17.578 | -35.72 | -43.48 | -46.73 |
| 17.656 | -35.26 | -43.26 | -46.62 |
| 17.734 | -34.84 | -42.99 | -46.51 |
| 17.813 | -34.48 | -42.67 | -46.41 |
| 17.891 | -34.17 | -42.36 | -46.28 |
| 17.969 | -33.85 | -42.05 | -46.11 |
| 18.047 | -33.51 | -41.75 | -45.92 |
| 18.125 | -33.12 | -41.46 | -45.72 |
| 18.203 | -32.65 | -41.19 | -45.50 |
| 18.281 | -32.16 | -40.93 | -45.28 |
| 18.359 | -31.64 | -40.64 | -45.06 |
| 18.438 | -31.17 | -40.31 | -44.83 |
| 18.516 | -30.76 | -39.94 | -44.59 |
| 18.594 | -30.44 | -39.54 | -44.34 |
| 18.672 | -30.21 | -39.16 | -44.18 |
| 18.750 | -30.35 | -38.81 | -44.21 |
| 18.828 | -30.89 | -38.60 | -44.42 |
| 18.906 | -32.03 | -38.57 | -44.75 |
| 18.984 | -33.80 | -38.86 | -45.17 |
| 19.063 | -35.54 | -39.51 | -45.62 |
| 19.141 | -37.17 | -40.33 | -46.07 |
| 19.219 | -38.55 | -41.30 | -46.47 |
| 19.297 | -39.66 | -42.19 | -46.80 |
| 19.375 | -40.30 | -42.97 | -47.02 |
| 19.453 | -40.45 | -43.51 | -47.07 |
| 19.531 | -40.07 | -43.77 | -47.00 |
| 19.609 | -39.14 | -43.68 | -46.88 |
| 19.688 | -38.16 | -43.22 | -46.75 |
| 19.766 | -37.21 | -42.68 | -46.57 |
| 19.844 | -36.18 | -42.11 | -46.37 |
| 19.922 | -35.07 | -41.62 | -46.19 |
| 20.000 | -33.99 | -41.23 | -46.02 |
| 20.078 | -32.96 | -40.84 | -45.90 |
| 20.156 | -32.26 | -40.44 | -45.89 |
| 20.234 | -31.92 | -40.13 | -45.96 |
| 20.313 | -31.87 | -39.93 | -46.08 |
| 20.391 | -32.11 | -39.92 | -46.22 |
| 20.469 | -32.47 | -40.12 | -46.39 |
| 20.547 | -32.92 | -40.38 | -46.56 |
| 20.625 | -33.37 | -40.68 | -46.73 |
| 20.703 | -33.79 | -41.03 | -46.92 |
| 20.781 | -34.14 | -41.44 | -47.13 |
| 20.859 | -34.42 | -41.90 | -47.38 |
| 20.938 | -34.66 | -42.39 | -47.61 |
| 21.016 | -34.86 | -42.82 | -47.83 |
| 21.094 | -35.01 | -43.19 | -48.06 |
| 21.172 | -35.10 | -43.57 | -48.32 |
| 21.250 | -35.23 | -43.95 | -48.61 |
| 21.328 | -35.39 | -44.31 | -48.91 |
| 21.406 | -35.59 | -44.66 | -49.21 |
| 21.484 | -35.81 | -45.01 | -49.55 |
| 21.563 | -36.00 | -45.37 | -49.91 |
| 21.641 | -36.15 | -45.74 | -50.27 |
| 21.719 | -36.36 | -46.12 | -50.63 |
| 21.797 | -36.64 | -46.46 | -51.00 |
| 21.875 | -36.94 | -46.76 | -51.41 |
| 21.953 | -37.24 | -47.08 | -51.84 |
| 22.031 | -37.50 | -47.45 | -52.25 |
| 22.109 | -37.72 | -47.90 | -52.63 |
| 22.188 | -37.94 | -48.45 | -52.99 |
| 22.266 | -38.18 | -49.01 | -53.31 |
| 22.344 | -38.39 | -49.56 | -53.62 |
| 22.422 | -38.56 | -50.05 | -53.95 |
| 22.500 | -38.77 | -50.47 | -54.34 |
| 22.578 | -39.04 | -50.91 | -54.75 |
| 22.656 | -39.31 | -51.39 | -55.12 |
| 22.734 | -39.58 | -51.88 | -55.44 |
| 22.813 | -39.80 | -52.37 | -55.71 |
| 22.891 | -39.98 | -52.80 | -55.98 |
| 22.969 | -40.16 | -53.16 | -56.26 |
| 23.047 | -40.36 | -53.53 | -56.56 |
| 23.125 | -40.53 | -53.91 | -56.84 |
| 23.203 | -40.65 | -54.31 | -57.06 |
| 23.281 | -40.69 | -54.72 | -57.18 |
| 23.359 | -40.61 | -55.05 | -57.19 |
| 23.438 | -40.44 | -55.29 | -57.11 |
| 23.516 | -40.19 | -55.41 | -56.97 |
| 23.594 | -39.94 | -55.40 | -56.77 |
| 23.672 | -39.72 | -55.23 | -56.55 |
| 23.750 | -39.51 | -54.89 | -56.33 |
| 23.828 | -39.33 | -54.52 | -56.12 |
| 23.906 | -39.15 | -54.15 | -55.90 |
| 23.984 | -38.98 | -53.77 | -55.66 |
| 24.063 | -38.79 | -53.38 | -55.40 |
| 24.141 | -38.58 | -52.94 | -55.15 |
| 24.219 | -38.28 | -52.43 | -54.93 |
| 24.297 | -37.88 | -51.95 | -54.73 |
| 24.375 | -37.45 | -51.55 | -54.55 |
| 24.453 | -37.00 | -51.16 | -54.37 |
| 24.531 | -36.57 | -50.78 | -54.19 |
| 24.609 | -36.17 | -50.40 | -54.00 |
| 24.688 | -35.76 | -50.00 | -53.81 |
| 24.766 | -35.32 | -49.64 | -53.60 |
| 24.844 | -34.81 | -49.30 | -53.38 |
| 24.922 | -33.85 | -48.93 | -52.86 |
| 25.000 | -33.27 | -48.23 | -52.73 |
